# Childhood cancer survivorship care: A qualitative study of healthcare providers’ professional preferences

**DOI:** 10.3389/fonc.2022.945911

**Published:** 2022-10-04

**Authors:** Jordana K. McLoone, Weihan Chen, Claire E. Wakefield, Karen Johnston, Rachael Bell, Elysia Thornton-Benko, Richard J. Cohn, Christina Signorelli

**Affiliations:** ^1^ Behavioural Sciences Unit, Kids Cancer Centre, Sydney Children’s Hospital, Randwick, NSW, Australia; ^2^ School of Clinical Medicine, University of New South Wales (UNSW) Medicine & Health, Discipline of Paediatrics, UNSW Sydney, Sydney, NSW, Australia; ^3^ Kids Cancer Centre, Sydney Children’s Hospital, Sydney, NSW, Australia

**Keywords:** Models of care, pediatric, pediatric oncology, survivorship, primary care, shared care

## Abstract

**Purpose:**

Childhood cancer survivorship care is a complex specialty, though it is increasingly being integrated into the general practitioner’s (GP) remit. Establishing the essential components of tertiary- and primary-led care, to maximize the benefits and overcome the challenges inherent to each, is essential to inform the development of survivor-centered, sustainable care models.

**Methods:**

We used the qualitative principles of semi-structured interviewing, verbatim transcription, coding (supported by NVivo12) and thematic analysis, to collect and evaluate the views and preferences of pediatric oncologists, survivorship nurse coordinators, and GPs currently caring for childhood cancer survivors.

**Results:**

Seventy healthcare providers (19 oncology staff and 51 GPs) from 11 tertiary hospitals and 51 primary practices across Australia and New Zealand participated. Participants reported specialist expertise and holistic family-centered care as the key benefits of tertiary and primary care respectively. Participants reported that tertiary-led survivorship care was significantly challenged by a lack of dedicated funding and costs/travel burden incurred by the survivor, whereas primary-led survivorship care was challenged by insufficient GP training and GPs’ reliance on oncologist-developed action plans to deliver guideline-based care. GPs also reported a need for ongoing access to survivorship expertise/consultants to support care decisions at critical times. The discharge of survivors into primary care limited late-effects data collection and the rapid implementation of novel research findings.

**Conclusions:**

Healthcare professionals report that while a risk-stratified, collaborative model of survivor-centered care is optimal, to be implemented successfully, greater provisions for the ongoing engagement of GPs and further access to GP education/training are needed.

## Introduction

Providing long-term, comprehensive cancer survivorship care to all childhood cancer survivors (CCS) is extremely Important given their lifelong risk of developing complex, chronic, and comorbid health concerns, including but not limited to cardiovascular, endocrine, and reproductive concerns, concerns related to the central nervous system such as learning disorders and epilepsy, as well as social, functional, and mental health concerns (1). While advancements are being made in the complex treatment of children with cancer (e.g. rapid advances in precision medicine (2, 3) and immunotherapy (4)) and services (e.g. telehealth), the challenge to provide optimal care continues to increase as more patients are surviving cancer and living longer lives and the high-needs CCS population continues to grow (5, 6). Particular complexity relates to providing more accessible and engaging care for the 68-81% of survivors who do not currently participate in long term follow-up care, due to personal barriers (such as travel costs, medical anxiety, or time constraints), systemic barriers (such as poor access and few survivorship clinics nationally), and age-related restrictions that limit access to their treating team once they have reached adulthood (7–9) A recent review of global childhood cancer survivorship care pathways indicates that almost all countries have difficulty managing this transition period and providing standardized care into adulthood (5).

Since the seminal report, “From cancer patient to cancer survivor: Lost in transition” was published in 2006 (10), greater research and resources have been focused on developing an optimal model of survivorship care (11). Almost 15 years later, there is a continued acknowledgement that there is unlikely to be one single, ideal model, as clinicians and researchers recognize that one size does not fit all - all survivors and all local settings have inherently varied resources and limitations. Many national cancer bodies now recommend risk-stratified pathways, involving oncologist and general practitioner (GP) involvement to varying degrees, as determined by the survivor’s age, the cancer treatment they received, their risk of recurrence, chronic health conditions, supportive care needs, and circumstances (12).

Within childhood cancer survivorship, models of care are not usually disease specific, but rather focus on providing late-effects expertise *via* a tertiary care model (including oncologist-led, nurse-led, or multidisciplinary models of care), a primary care model (GP-led), or a shared care model (which envisages both tertiary and primary clinical teams working collaboratively) (13). The Journal of Cancer Survivorship recently called for further research focused on tailored models of care to elucidate exactly who, where, and for which survivors particular models are optimal, acknowledging that it is unlikely for comprehensive cancer care to be delivered by one type of specialist exclusively (14).

Given that oncologists, nurse specialists, and GPs all play pivotal roles in the provision of survivorship care, we sought to better understand their views on hospital-based, GP-based, and shared-care models. We sought to explore health professionals’ views on the limitations of these models in their current form, as well as what they consider to be critical to the successful implementation of feasible, sustainable, engaging and equitable life-long survivorship care.

## Materials and methods

The Australian and New Zealand Children’s Hematology and Oncology Group (ANZCHOG) Survivorship Study (15) surveyed childhood cancer survivors and oncology staff from every tertiary, paediatric oncology treatment centre in Australia and New Zealand (representing 11 children’s hospital). Oncology staff were invited to participate if they were the Head oncologist or the clinical nurse consultant (or equivalent) of the survivorship clinic within their hospital. Survivorship oncologists and nurse specialists were invited to participate in this study *via* a mailed letter and follow-up phone call. Survivors participating in the ANZCHOG Survivorship Study were also asked to nominate their current GP, who was then contacted by the research team *via* a mailed invitation letter, and invited to participate in the study.

A multidisciplinary team, including a clinical oncologist, psychologist, survivorship nurse, behavioural scientist, GP, and hospital director, developed two semi-structured interview guides. One guide was to interview oncology staff and the other to interview GPs. The oncology staff interview explored survivorship care practices within the hospital setting (e.g. clinic information, eligibility, guideline adherence), transition pathways, views on models of care, resource needs, challenges and potential solutions. The GP interview explored survivorship care practices within the primary care setting (e.g. GPs’ confidence, knowledge, and communication with oncology teams), and GPs’ views on models of care, resource needs, challenges and potential solutions. GP demographic data, including gender, age, years of practice, number of survivors in their practice and postcode of their practice location was collected during the interview.

First author JM (PhD) has over a decade in qualitative research practice and conducted all oncology staff interviews, while a PhD qualified research assistant completed the GP interviews. JM was known to some oncology staff, as an active member within the Australian paediatric oncology survivorship community. The interviewer had no relationship to GP participants. Interviews were conducted in private, were audio recorded, field notes were made and recordings were transcribed verbatim. Analysis was guided by the Braun and Clarke approach (16). Authors JM and WC read all interviews for familiarity with their content. The coding heirarchy was developed based on these initial readings. Interview data were then coded line by line and preliminary themes were organised to establish central core ideas. The South-Eastern Sydney Local Health District granted ethical approval (Reference: 12/173).

## Results

We interviewed the lead pediatric oncologist at each of the ANZ tertiary survivorship clinics (n=9) and their lead survivorship nurse (typically a clinical nurse consultant) (n=10) (noting that two oncologists led two clinics each and one nurse led two clinics). Fourteen participants worked in an Australian hospital and five in a New Zealand hospital, average interview length was 34 minutes. Fifty-one GPs were also interviewed, average interview length was 19 minutes. See **Table 1** for participants’ demographic details. Of the 19 invited oncology staff, 19 participated (100% response rate); out of the 160 invited Australian GPs, 74 opted to participate (46% response rate), but data saturation was achieved after 51 interviews, so no further interviews were scheduled. We considered data saturation to have been achieved at the time when subsequent interviews were no longer contributing additional or new information.

**Table 1:** Demographic information of GPs and oncology staff.

Demographics	GPs (n=51)	Oncology staff (n=19)
Female	22 (43.1%)	13 (68.4%)
Mean years of practice (SD)	28.25 (12.2)	N/A
Mean number of CCSs cared for (SD)	2.16 (1.73)	N/A
Country of practice		
Australia	51	14
New Zealand	0	5
ARIA Class of practice		
Major city	28	19
Inner regional	9	0
Outer regional	2	0

CCS, childhood cancer survivor; ARIA, accessibility/remoteness index of Australia calculated using practice postcodes (17), the distribution of respondents is reflective of the geographic distribution of GPs practicing in Australia; GP, general practitioner; SD, standard deviation; N/A, not assessed.

Several themes were identified during analysis of the HCP interviews. These central themes are reported below.

### The importance of expert multidisciplinary care

Oncologists and GPs shared their ongoing support for traditional, hospital-based case management with multidisciplinary team (MDT) involvement, as they believed this offered the highest level of expertise and knowledge available to survivors. *“*[Hospital-based care offers] *particular knowledge and expertise in post-cancer treatment and late effects”* (male, oncologist). Participants reported that oncologists, with survivorship expertise, were more likely to rapidly incorporate new research findings and advances in the field into the care they provided. They reported that oncologist specialists were often more aware of the broad ranging issues survivors faced, such as mental health concerns, financial toxicity, and educational or career impacts, and offered clear pathways to relevant support networks available within the survivor’s community.

Hospital-based, MDT care was also viewed as a convenient “*one stop shop*” where survivors could receive a holistic review and care for many of their comorbidities concurrently. Oncology staff reported preferences for greater involvement of multidisciplinary medical specialists (e.g. neurology, adolescent medicine, onco-fertility) and allied health professionals (e.g. dietitians, physiotherapists, disability service coordinators) within their survivorship clinics. However, while more staff were desired, oncology staff expressed their difficulty in securing enough protected time even for their own staff to contribute to survivorship clinics, over and above their clinical roles.

*“…lack of funding, lack of resources, lack of space … lack of staff … lack of protected time … there’s only a finite number of people we can see each year.”* (female, clinical nurse consultant (CNC))

### Managing survivors’ unique needs over time

Despite strong support for expert, oncology-led care with involvement from a broad range of specialties, it was also widely acknowledged that survivors had unique risk profiles, surveillance, and care needs and that this changed over the course of the survivorship period.

In terms of unique risk profiles and needs, participants highlighted the importance of risk-stratification to improve the efficacy of survivorship care delivery.

*“You could definitely have triage clinics to determine who would go where, you could base a lot of it on diagnostic risk stratification”* (female, CNC)

In terms of survivors’ unique needs over time, it was suggested that the existing adult survivorship care infrastructure could be leveraged to also accommodate adult survivors of childhood cancer.

*“…as survivorship develops as a significant entity amongst adult patients … there may be an opportunity to obtain synergy with adult clinics with better understanding of the issues of long-term survivors.”* (male, oncologist)

However, while transitioning pediatric survivors to adult survivorship clinics was viewed as a possible solution in the longer term, there remained a current lack of transition services, causing a reluctance to discharge survivors as “*there’s nowhere to put them.*” (female, CNC)

*“There’s reluctance* [for] *some adult facilities to take* [childhood cancer survivors] *on, there’s a lack of clarity about where’s the best place to send them.”* (female, CNC)

### Bridging the gap between tertiary and primary care

There was a general consensus that either an experienced survivorship nurse, or a community GP, could manage low-to-medium risk survivors’ care *“…with feedback to late effects clinics in critical times”* (male, oncologist). A large proportion of oncology staff acknowledged the potential benefits of nurse-led survivorship care, with survivorship nurses coordinating survivors’ care by liaising with a multidisciplinary team and the survivors’ nominated GP.

*“From a funding … and logistical perspective, I like the idea of the nurse-led clinic with that person liaising with … community based, or hospital based* [providers]*.”* (female, oncologist)

Oncologists unanimously acknowledged the importance of involving GPs in follow-up care to *“function as the liaison for the transition out into the community”* (male, oncologist), while emphasizing that *“the treating center … would always have to have some level of involvement”* (female, CNC).

Oncology staff recognized that a shared-care model between hospital and primary care practices required improved communication and closure of the “feedback loop” between specialists and GPs. Oncologists continued to want to receive progress and updates on survivors’ health outcomes and their recommended surveillance schedules from GPs. Similarly, GPs wanted greater involvement throughout the course of their patient’s cancer treatment so that they were in a well-informed position to continue care during survivorship. Oncology staff perceived that GPs struggled with this lack of continuity, *“…we’ve taken over the care of the patient and families for … years and then we go ‘alright we’re done with you, back to the GP’…* [but] *communication in the interim is not always ideal, families move.”* (female, CNC). Lack of communication and collaboration with treating teams during treatment left GPs feeling that they lacked insight into their patient’s cancer care and how best to continue to deliver care in a collaborative manner.

*“I don’t have any direct contact with the hospital. I don’t know how it works … I don’t know any of the team … That’s a huge disadvantage.”* (male, GP, practicing 27 years)

Barriers to improved bi-directional inter-practice communication included a lack of administrative staff to manage scheduling and correspondence, as well as data managers to oversee record-keeping between sites. Survivorship care plans were reported as labor intensive and their dissemination was *“dependent on workload”* (female, oncologist). The importance of the survivorship nurse role in potentially coordinating much of this work was discussed.

*“If you’ve got a specialized nurse that’s keeping an eye on everything and … on the communication between specialists, GPs, the nurses and the patient, that’d be very good…”* (female, GP, practicing 10 years)

### The perceived benefits of receiving care in the community

Though hospital-community collaboration was viewed as challenging, receiving local, GP-led care was seen to overcome the many logistical and financial issues families faced when trying to access tertiary-led care, which is only located in major urban cities.

*“When the family goes up to Sydney … it costs a fortune to stay up there and* [they] *can’t afford it”* (male, GP, practicing 39 years),

Some GPs felt confident that they could lead survivors’ care, noting that their strong counselling practices were ideal for providing *“patient-centered care”* (female, GP, practicing 35 years) and mental health support. Additionally, they reported that the *“traditional family model”* (male, GP, practicing 43 years) within GP services provided a sense of long-term continuity and the opportunity for whole-family care, noting the impact of childhood cancer on the whole family.

*“Patients are very familiar with* [long-term practice managers] … *it’s not uncommon for patients to come back after years …* [we] *pull up the old records and roughly know what it’s going to be about* [and] *just book them in…”* (male, GP, practicing 43 years)

### The perceived challenges of receiving care in the community

Both oncology staff and many GPs reported low confidence in local GPs’ knowledge of survivorship care, as childhood cancer *“is a relatively rare condition”* and there is a *“big lack of knowledge and education”* (female, GP, practicing 14 years) surrounding survivorship issues within the primary sector. GPs also perceived survivors’ lack of confidence and trust in their care.

*“I don’t think that they [the survivor] would have a huge degree of trust - nor would I blame them - in the quality of care offered by the GP.”* (male, GP, practicing 27 years)

As such, GPs were mostly willing to provide survivorship care for low-risk survivors, with higher risk cases and concerns directed to specialists.

*“When these children have a high intensity illness, families get very reliant on* [and] *comfortable with the treating specialist. So sometimes their questions are satisfactorily answered at that level.”* (male, GP, practicing 25 years)

GPs noted that survivors more often sought medical assistance from them regarding more routine adult health-related issues and that this led to skewed perceptions that survivors have limited risk of developing late-effects. In addition, GPs reported that the large patient volumes seen by GP services acted as a competing interest to furthering childhood cancer survivorship care education, which constituted only a very small proportion of their case load.

*“When you’re seeing 200 patients a week, I only have enough time and resources to react to what’s coming in through the front door.”* (male, GP, practicing 27 years).

*“…the average GP in their lifetime might* [probably] *look after one or two* [childhood cancer survivors] … *It’s not something that’s top on our list of getting really good at”* (male, GP, practicing 32 years).

GPs reported that, as appropriate remuneration to provide comprehensive survivorship care within their practice was not available, this limited any economic justification for up-skilling.

*“You’re asking people who are basically in semi-sweatshop conditions to do a high-level function when neither the patient nor the community want to fund that.”* (male, GP, practicing 30 years)

Consequently, GPs reported that they often took a more reactive rather than proactive approach to late effects surveillance and highlighted GPs’ need for treatment-specific details, as well as *“evidence-based and guideline-driven”* (female, GP, practicing 14 years) surveillance recommendations, from oncologists at the beginning of the treatment phase.

*“In the actual stage of the disease … the specialist should pass on as much information as possible* [to GPs]*, for then* [GPs] *will know what to expect 10 years, 20 years down the line…”* (male, GP, practicing 21 years)

### The need for expert recommendations to guide general practitioners

Continuing onward from the treatment stage, GPs noted a prescriptive approach during survivorship was crucial to accurately counsel survivors, clarify responsibilities, and review and formulate management plans. GPs emphasized that information and communication should be succinct, with specific action plans.

*“*[GPs] *are inundated with information from all quarters … when I get any discharge summary … I only focus on*, [the section] *‘GP to action’.”* (male, GP, practicing 27 years)

In addition, clear contact information, referral pathways, and the ongoing availability of oncology staff was also reported as crucial for resolving queries and making referrals back to hospitals.

*“*[This] *allows me to ring them up … if I have a detailed question. Then if I need to refer him back, it’s not … out of the blue.…I think it does provide a valuable support for* [GPs] *definitely.”* (male, GP, practicing 12 years)

### Future improvements

Overall, health professionals acknowledged that survivorship care is *“so variable, depending on individual cases”* (female, GP, practicing 12 years), suggesting that future improvements in survivorship care should allow care to be tailored according to survivors’ individual needs rather than being standardized.

*“I don’t think it would be very helpful to have just a blanket thing that you do for all of them, because you’d be wasting their time.”* (female, GP, practicing 21 years)

GPs suggested creating a specific Medicare item number (i.e. a specific, billable Medicare service subsidized by the Australian government) for childhood cancer survivors to encourage more GP services to provide higher quality survivorship care.

*“[Survivorship care] could actually be time-consuming … GPs are paid on a person-in-the-room basis … if* [the extra time] *was remunerated then you’d certainly get a much better uptake in the community of that role.”* (female, GP, practicing 8 years)

Australian participants supported the creation of a national database to hold survivor information (for example, diagnostic, treatment, and late effects data). In NZ, where a national database already exists, participants felt that it was not being used to its full potential to determine the prevalence and risk factors for later disease and morbidity, especially among high-risk and rare childhood cancers.

*“The database that we’re using is national, the format of the health passport is national. Where we haven’t progressed further is with doing and looking at what we’ve found and that’s an another something we need money for, we need time, because you know we’ve [only] got impressions* [not evidence].” (female, oncologist)

Further national collaboration was reported as having the potential to support and guide the standardization of care guidelines and survivorship care plans. Oncology staff also noted the benefits of using nationally collated information to develop centralized education materials for GPs and survivors across sites. Additionally, they suggested a more collaborative approach in survivorship research could improve the representativeness of the sample, prevent research replication and overlap, and maximize the use of funding.

*“…people* [need] *to collaborate to develop databases to ensure that any research coming out of Australia represents a national database and is not just single or a couple of hospitals collaborating…”* (male, oncologist)

## Discussion

Oncology staff and GPs were generally aligned in their perceptions of the various benefits and challenges of delivering high quality childhood cancer survivorship care. The time and cost it takes to deliver complex care, encompassing risk-based surveillance, the management of late-effect comorbidities, and the recognized psychosocial impacts associated with a diagnosis of childhood cancer, were perceived as extremely challenging or unsustainable. Risk-stratified care was seen as ideal, with both oncology and general practices collaborating to varying degrees as the survivor traversed the survivorship period. Yet this model was challenged by the high level of communication, instruction, and data sharing that this would require between multiple practitioners, across several decades of survivorship. Furthermore, any form of shared care, or risk-stratified discharge of low-medium risk survivors to primary care would require the provision of greater training for GPs, who consistently report within this study and elsewhere in the literature (18, 19), low confidence to provide up-to-date care for childhood cancer survivors (20). Given that GPs in this study reported caring, on average, for two survivors across a career of 28 years, it is understandable that they may lack the experience and motivation to invest time in training in this field. Greater liaison with, and education delivered by survivorship nurses, or the use of new innovations in training are possible solutions. GP training should be targeted at GPs currently caring for survivors and new research is needed to develop and evaluate high quality, purpose-built, online, brief, content dense eLearning videos, developed specifically for busy GPs.

The potential for specialist nurses to act as liaisons, bridging the gap, coordinating services, facilitating transitions and educating GPs was proffered in this study and has been reported in the previous literature (21). There is likely room for substantial growth in this area and the potential for experienced nurses to lead the development of resource-limited clinics is strong (20). Funding and investment in the next generation of expert nurses to support such positions remains critical, as does further research to evaluate the efficacy of such models of care.

The issue of insufficient funding has been consistently reported as a barrier in coordinating survivorship care in this study and in existing literature (6, 22, 23). Various strategies were offered, including increasing the responsibility of lower-cost nurses (comparative to higher-cost oncologists or a full multi-disciplinary team), and the creation of specific Medicare Benefit Schedule item numbers (funded by the Federal Government) that would allow hospitals and GPs to claim greater funding/remuneration. Despite the varying healthcare billing systems and practices internationally, the aforementioned strategies could be implemented and modified according to locally specific remuneration issues.

Survivorship care plans (which provide information about a survivor’s cancer treatment, follow-up care needs, surveillance schedules and health education) were also discussed as a means to increase communication between oncologists and GPs and to provide GPs with specialist recommendations that could be implemented at the local level, reiterating existing literature (24, 25). However, survivorship care plans have demonstrated variable efficacy to date and limited increases in adherence to surveillance recommendations, detection of late effects and prompt referral (13, 26, 27). While our study reports that GPs would prefer that oncologists provide highly prescriptive care plans early on, most GPs typically report not receiving a care plan (25), and oncologists recognized that these were disseminated only as resources allowed, with issues surrounding the labor intensity of preparing survivorship care plans.

It is important to continue to reassess old challenges in light of new solutions. Post COVID-19, a more general reimagining of care delivery, more reliant on telehealth seems possible (28). Distance-delivered care pathways and survivorship care plans can now be supported by smart platforms, computerized algorithms and new technology, reducing some of the time/cost limitations that have been traditional barriers (29) (30, 31). There is strong potential to develop more cost efficient and more equitable care solutions, especially for regional and rural families *via* evolving telehealth or e-health programs. Future research is needed to provide accurate effectiveness and cost data, including costs to the survivor, provider and health system. The feasibility and acceptability of distance-delivered interventions to support survivorship care has been reported (32), but further evaluation of larger trials, with long-term outcome data is called for.

### Study limitations

Participants in our study were selected for their expertise and prior experience in childhood cancer survivorship care and therefore may not represent the views of all health professionals within the field. Despite potential contextual differences between various countries, our findings surrounding providers’ preferences are consistent with internationally reported research findings (5) and can be used to supplement and inform future strategies to enhance the delivery of childhood cancer survivorship care globally. There remains an ongoing need for longitudinal studies evaluating changes in providers’ preferences over time to keep pace with advancements in the medical field and rapidly changing circumstances within healthcare systems and technology.

## Conclusion

The optimal delivery of sustainable care requires input from multiple health professionals and is unlikely to remain static across the entire survivorship period. As such, communication between key providers including oncologists, survivorship nurses, and GPs, needs to be maintained from diagnosis to end of life. Investment in strategies to support collaboration is necessary or else risk-stratified care will remain impeded. Funding for roles to manage communication, points of transition, effective survivorship care plan dissemination and data sharing is required, as is further research to develop and evaluate such initiatives.

## Data availability statement

The datasets presented in this article are not readily available to protect participant and patient confidentiality. Requests to access the datasets should be directed to the corresponding author and will only be shared in accordance with the ethical consent provided by participants on the use of confidential/identifiable human data. Requests to access the datasets should be directed to j.mcloone@unsw.edu.au.

## Ethics statement

The studies involving human participants were reviewed and approved by the South Eastern Sydney Local Health District. The patients/participants provided their written informed consent to participate in this study.

## Author contributions

JM (first author) contributed to the study conception and design, recruitment, interviewing, analysis and manuscript writing. WC contributed to data analysis and manuscript writing. CW contributed to the study conception and design, and manuscript revision, read and approved the submitted version. KJ contributed to design and contributed to manuscript revision, and read and approved the submitted version. RB contributed to design and contributed to manuscript revision, and read and approved the submitted version. ET-B contributed to design and contributed to manuscript revision, and read and approved the submitted version. RC, contributed to the study conception and design and manuscript revision, and read and approved the submitted version. CS contributed to the study conception, recruitment and design and manuscript revision, and read and approved the submitted version.

## Funding

The Behavioural Sciences Unit (BSU) is proudly supported by the Kids with Cancer Foundation. JM is supported by the Medical Research Future Fund, Australian Brain Cancer Mission, Brain Cancer Survivorship Grant (MRFBC000002). CW is supported by the National Health and Medical Research Council of Australia (APP1143767 and RG2008300). CS is supported by a Cancer Institute NSW Early Career Research Fellowship (2020/ECF1144). This work has been supported by the Kids Cancer Alliance and a Medical Research Future Fund (MRFF) Brain Cancer Survivorship grant (MRFBC000002).

## Acknowledgments

We would like to thank Joseph Alchin for research assistance.

## Conflict of interest

The authors declare that the research was conducted in the absence of any commercial or financial relationships that could be construed as a potential conflict of interest.

## Publisher’s note

All claims expressed in this article are solely those of the authors and do not necessarily represent those of their affiliated organizations, or those of the publisher, the editors and the reviewers. Any product that may be evaluated in this article, or claim that may be made by its manufacturer, is not guaranteed or endorsed by the publisher.
